# The role of internalised HIV stigma in disclosure of maternal HIV serostatus to children perinatally HIV‐exposed but uninfected: a prospective study in the United States

**DOI:** 10.1002/jia2.26167

**Published:** 2023-11-01

**Authors:** Mariam Davtyan, Deborah Kacanek, Jessica Lee, Claire Berman, Ellen G. Chadwick, Renee Smith, Liz Salomon, Toinette Frederick, for the Pediatric HIV/AIDS Cohort Study

**Affiliations:** ^1^ Department of Pediatrics Keck School of Medicine University of Southern California Los Angeles California USA; ^2^ Harvard T.H. Chan School of Public Health Center for Biostatistics in AIDS Research Boston Massachusetts USA; ^3^ Department of Epidemiology Harvard T.H. Chan School of Public Health Boston Massachusetts USA; ^4^ Department of Pediatrics Feinberg School of Medicine Northwestern University Chicago Illinois USA; ^5^ Department of Pediatrics College of Medicine University of Illinois Chicago Illinois USA

**Keywords:** internalised HIV stigma, PHACS, SMARTT, maternal HIV disclosure, parental HIV disclosure, perinatal HIV exposure

## Abstract

**Introduction:**

Decisions to disclose HIV serostatus may be complicated by internalised HIV stigma. We evaluated the association of internalised HIV stigma in biological mothers living with HIV with disclosure of their serostatus to their children perinatally HIV‐exposed but uninfected (CHEU).

**Methods:**

Mothers and their CHEU were enrolled in the United States (U.S.)‐based Surveillance Monitoring for Antiretroviral Therapy (ART) Toxicities (SMARTT) study of the Pediatric HIV/AIDS Cohort Study (PHACS), a longitudinal study of outcomes related to *in utero* exposure to HIV and ART among CHEU. Mothers completing at least one stigma and disclosure assessment starting at the child's age 11‐, 13‐, 15‐ and/or 17‐year study visits between 16 August 2016 and 1 October 2020 were eligible. Stigma was measured with the 28‐item Internalised HIV Stigma Scale (IHSS). Mean stigma scores were linearly transformed to a range of 0–100, with higher scores indicating greater levels of stigma. At each visit, mothers were asked if their child was aware of their HIV diagnosis and at what age the child became aware. The Kaplan‐Meier estimator evaluated the cumulative probability of disclosure at each child age. Logistic regression models with generalised estimating equations to account for repeated measures were fit to examine the association between stigma and disclosure, controlling for relevant socio‐demographic variables.

**Results:**

Included were 438 mothers of 576 children (mean age 41.5 years, 60% U.S.‐born, 60% Black/African American and 37% with household income ≤$10,000). The prevalence of disclosure across all visits was 29%. Mothers whose children were aware versus not aware of their serostatus reported lower mean IHSS scores (38.2 vs. 45.6, respectively). The cumulative proportion of disclosure by age 11 was 18.4% (95% CI: 15.5%, 21.8%) and 41% by age 17 (95% CI: 35.2%, 47.4%). At all child ages, disclosure was higher among children of U.S.‐born versus non‐U.S.‐born mothers. After adjusting for age, marital status and years since HIV diagnosis, higher IHSS scores were associated with lower odds of disclosure (OR = 0.985, 95% CI: 0.975, 0.995).

**Conclusions:**

Providing support to women as they make decisions about serostatus disclosure to their children may entail addressing internalised HIV stigma and consideration of community‐level factors, particularly for non‐U.S.‐born mothers.

## 1 INTRODUCTION

HIV‐related stigma involves negative, judgemental or discriminatory attitudes that people and society have about persons living with HIV (PLWH) and it has been a long‐established barrier to optimal HIV care and service uptake [1]. It has also been linked to decreased social support, adverse mental health conditions and marginalisation, resulting in PLWH being excluded from exerting influence on dynamic structures and systems (i.e. healthcare, political, economic and community) [1, 2, 3, 4, 5].

HIV stigma has also been linked to reduced HIV serostatus disclosure to others [6]. HIV serostatus disclosure entails informing others of one's HIV status and is a complex process by which personal information is communicated [7]. Disclosure occurs on a continuum, from non‐disclosure to partial disclosure, to full disclosure, and involves multiple cognitive, emotional and behavioural reactions [8, 9, 10].

Disclosure of parental HIV serostatus to children has been documented as a significant challenge for parents [8, 11]. Parental serostatus disclosure to children has implications for parental and child health, parenting and guardianship plans, increased risk of unintended disclosures and discrimination against other family members, and family dynamics [8]. Children are typically disclosed to less often, compared to intimate partners, other family members and healthcare providers [11, 12]. Research shows that rates of parental (mothers and fathers) disclosure to children range from 20% to 97% in studies conducted within the United States (U.S.) and between 11% and 44% in studies conducted outside of the U.S [8]. Variations in disclosure proportions across different studies have been attributed in part to differences in sample characteristics and the lack of standardisation in how disclosure is measured [8].

Other studies have shown that the proportion of mothers disclosing their HIV serostatus to their children range from 30% to 66% [13, 14]. Noted reasons for maternal disclosure have included wanting to tell the child herself, the child's right to know, preparing the child for the future, a sense of parental obligation and the need to protect the child [11, 15]. Reasons mothers choose not to disclose may include HIV stigma and discrimination, not wanting to burden the child and perceived immaturity of the child [11, 15, 16, 17].

Maternal benefits of disclosure to children may include stress alleviation around the need to hide medical care, better compliance with clinical appointments, lower anxiety and depression, higher levels of social support, stronger relationships with children and greater family cohesion [12, 17, 18, 19, 20, 21, 22, 23, 24]. However, disclosure to children may also lead to fears about the quality of the relationship with their child declining and feeling overwhelmed and nervous post disclosure [20, 25].

Benefits of disclosure for children may include decreased problematic behaviours over time, reduction in negative mood and depression, increase in household responsibilities and higher self‐concept [24, 26, 27]. Downsides of the disclosure may encompass lower emotional and social functioning, externalizing symptoms, higher levels of depression, unprotected sexual behaviours and substance use, and negative feelings about and poor performance in school [19, 28, 29, 30, 31].

The contribution of HIV stigma to decisions about HIV serostatus disclosure to others has also been explored in the literature. Higher levels of internalised HIV stigma, defined as stigma (i.e. perceived, experienced, enacted and anticipated) that is incorporated into the self‐definition, results in negative self‐perception and self‐injurious behaviours, and lower levels of disclosure [5, 32, 33, 34, 35, 36]. For parents living with HIV, fear of discrimination due to secondary disclosure by children, rejection and loss of respect from children, and internalised HIV stigma manifested as self‐shaming and experienced guilt about having HIV, have all been implicated as barriers to disclosure [37, 38, 39, 40]. The explicit role of maternal internalised HIV stigma in serostatus disclosure to their children, particularly children perinatally HIV‐exposed but uninfected (CHEU) who may need long‐term monitoring, is currently understudied. Understanding the role of internalised HIV stigma in disclosure among mothers and their CHEU may help women with their disclosure decisions [15, 16, 17, 21, 24, 27, 41].

In this study, we aimed to understand the role of internalised HIV stigma in the disclosure of maternal HIV serostatus to their CHEU (ages 0–17 years) enrolled in the Surveillance Monitoring for Antiretroviral Therapy (ART) Toxicities (SMARTT), a study of the Pediatric HIV/AIDS Cohort Study (PHACS). Specifically, we estimated the prevalence of disclosure to CHEU and determined the association between internalised HIV stigma and disclosure.

## 2 METHODS

### 2.1 Ethical considerations

The SMARTT study was reviewed and approved by Institutional Review Boards (IRBs) at all participating sites and Harvard T.H. Chan School of Public Health. Mothers provided informed consent for themselves and their children to participate in the PHACS SMARTT study. Children signed an age‐appropriate (per site IRB guidelines) assent form to participate in a study about their general health and development. Children who turned 18 years during the study period were invited to participate in the SMARTT Young Adult Cohort only if they were aware of their mother's HIV serostatus and their own HIV and ART exposure.

### 2.2 Study population

SMARTT evaluates the long‐term safety of ART taken during pregnancy among women living with HIV and their CHEU and its methods have been described elsewhere [42]. The population for this study included biological mothers of CHEU enrolled in the Static and Dynamic cohorts of SMARTT as of 1 October 2020. Mothers were eligible if they had at least one study visit since 16 August 2016, when SMARTT began collecting interview data on internalised HIV stigma and maternal serostatus disclosure to children at the child's visit ages 11, 13, 15 and 17 years.

### 2.3 Exposure measure

The primary exposure was internalised HIV stigma, measured with the 28‐item validated Internalised HIV Stigma Scale (IHSS) [43]. The IHSS measures current internalised HIV stigma and consists of four subscales: stereotypes (12‐items), disclosure concerns (5‐items), social relationships (7‐items) and self‐acceptance (4‐items), with each item response ranging from 0 to 4. To obtain an overall stigma score, mean scores of the IHSS subscales were averaged and linearly transformed to a range of 0–100, with higher scores indicating greater perceptions and experiences of internalised HIV stigma. Participants were allowed up to a 1‐year window around the visit age to complete the corresponding stigma surveys to be included in the analysis. Stigma surveys with at least half of the items in each subscale answered were considered complete. The rationale for only including participants who responded to at least half of each subscale was that some of the IHSS subscales had very few items compared to the others. The IHSS score comprised the average of the completed subscale items.

### 2.4 Outcome measure

The primary outcome was maternal HIV disclosure status. Disclosure status was assessed via interview at child visit ages 11, 13, 15 and 17 years and defined as a “yes” response to the question “Is [child's name] aware of your HIV diagnosis?” This disclosure could have been by the child's mother or by someone else. To estimate the probability of disclosure by each child age, the actual age (in years) at which the child was reported to have learned of the mother's HIV serostatus was used (assessed with the question “How old was [child's name] when he/she first learned you had HIV?”). In the event of a discrepancy in actual age across interviews for the same child, the age reported at the first interview in which the mother reported the child was aware of her diagnosis was used.

### 2.5 Covariates

Child and maternal covariates were also considered. Child characteristics included cohort (Static/Dynamic), age, sex, race, ethnicity, educational level and primary language. Maternal characteristics included site/region, country of birth, age at time of stigma/disclosure assessment, race, ethnicity, language spoken at home, highest educational level, annual household income, employment status, living arrangement, marital status, health limitations (i.e. difficulties with mobility, physical activity, work, household chores, etc.) and years since HIV diagnosis. In the adjusted model of the association between internalised HIV stigma and maternal disclosure, maternal age, marital status and years since maternal HIV diagnosis were considered as potential confounders.

### 2.6 Statistical analysis

The prevalence of disclosure in the study population and by child age at the time of assessment was estimated from all visits. The denominator included everyone with a visit at that child age, regardless of disclosure status, and the numerator was the number of children who were aware of their mother's HIV serostatus as of that age. The way the child learned of the mother's diagnosis and reasons for disclosing or not disclosing were also summarised from the first interview that each child was reported to have been aware of their mother's HIV serostatus. Among children who were never reported to be aware of their mothers’ HIV serostatus, the distribution of maternal intention to disclose in the future as reported at the first visit when disclosure and stigma were assessed was summarised, and reasons for not disclosing were summarised by maternal intention to disclose. Distributions of child and maternal demographic, socio‐economic and clinical characteristics were summarised and compared at visits at which the mother reported the child being aware of her diagnosis versus not aware. Distributions were also summarised by child visit age.

The Kaplan‐Meier estimator was used to estimate the probability of disclosure by each child age, using the actual age at which the mother reported the child learned of her HIV diagnosis. Children were censored at the age at which disclosure was reported, or, if not disclosed to, at the age at time of the last visit when their mothers were interviewed. The estimated probability of disclosure by each child age was compared between mothers who were born in the U.S. and Puerto Rico and mothers born in other countries. A score test from Cox proportional hazards models with the robust sandwich estimator was used to evaluate whether the Kaplan‐Meier curves were statistically different at any time point, accounting for correlation from repeated measures (multiple children per biological mother). The proportionality assumption of the Cox proportional hazards model was tested via the Kaplan‐Meier curves graphically and the Schoenfeld residuals.

To evaluate the association between internalised HIV stigma and maternal HIV disclosure status, univariable and multivariable logistic regression models were fit with generalised estimating equations to account for repeated measures per mother and child. Both unadjusted and adjusted measures of association were evaluated with odds ratios (ORs) and 95% confidence intervals. All analyses were performed using SAS software, Version 9.4.

## 3 RESULTS

### 3.1 Study population

**Figure 1:**
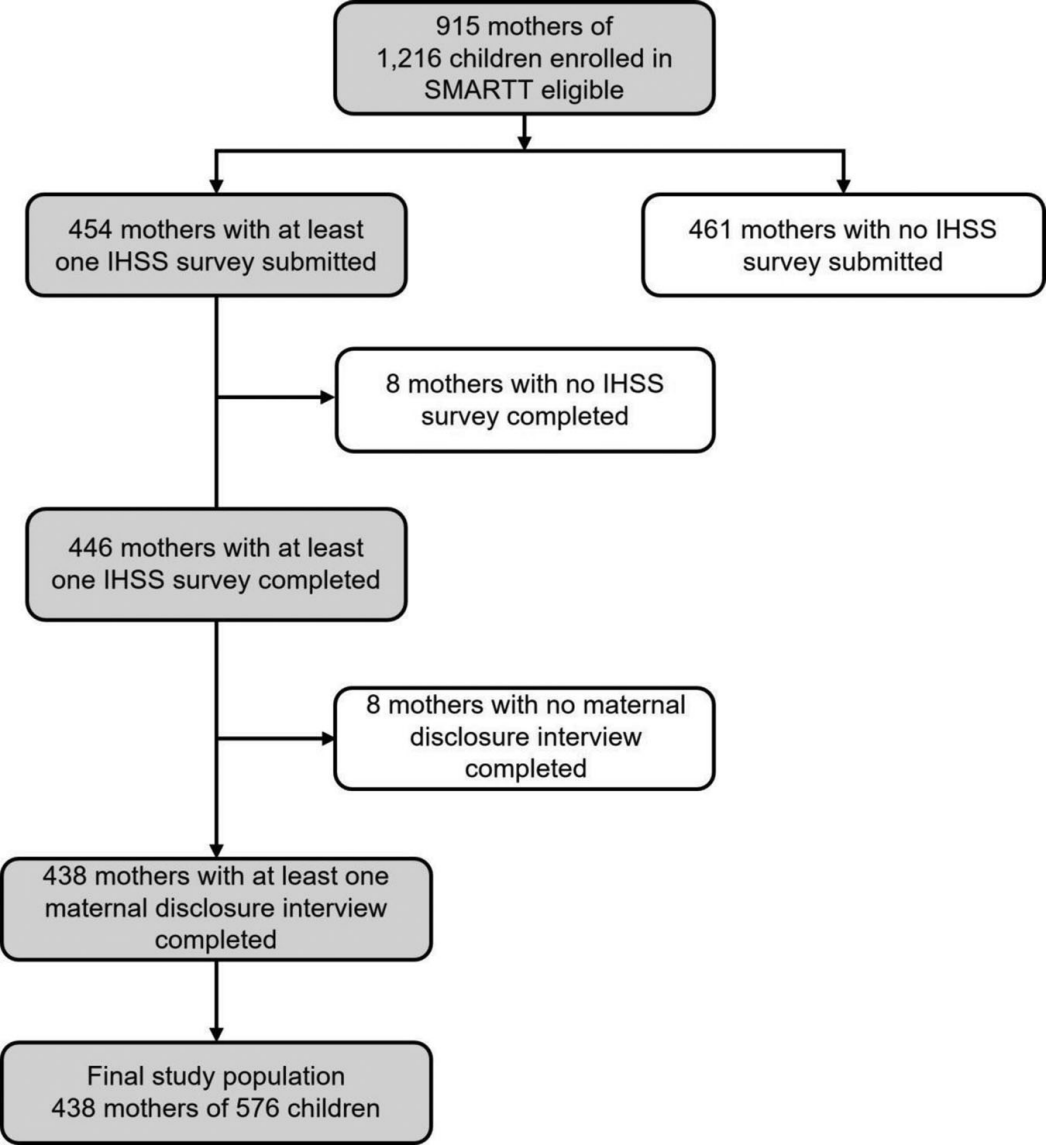
Study population selection. Abbreviations: SMARTT, Surveillance Monitoring for Antiretroviral Therapy Toxicities; IHSS, Internalised HIV Stigma Scale.

As of 1 October 2020, 915 biological mothers of 1216 children were eligible for the analysis. Of the 915 mothers, 454 (49.6%) submitted at least one IHSS survey, while 461(50.4%) mothers had no survey submitted. Of those who submitted their surveys, 446 mothers (48.7% of the 915 eligible) submitted a completed IHSS. Among the 446 mothers with a completed IHHS, eight did not complete the maternal disclosure interview, leaving a total of 438 mothers of 576 children across 739 visits included in the analysis (Figure 1).

### 3.2 Prevalence of maternal HIV serostatus disclosure

**Figure 2:**
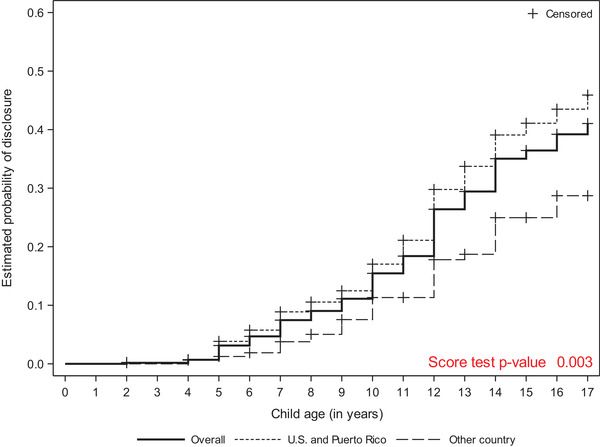
Estimated probability of disclosure by mother's birth region across child's age.

Mothers indicated that the child was aware of their HIV serostatus at 28.1% (208/739) visits, representing 166 unique children and 132 unique mothers (28.8% of 576 children and 30.1% of 438 mothers in the final study population). Prevalence of disclosure was lowest at the child age 11 visit (11%) and increased to 28% at the age 13 visit, 42% at the age 15 visit and 47% by the age 17 visit. The estimated probability that a child was aware of their mother's serostatus by age 11 was 18.4% (95% CI: 15.5%, 21.8%), and this probability increased to 41% (95% CI: 35.2%, 47.4%) by age 17. Additionally, there was a higher probability of disclosing to the child among mothers born in the U.S. and Puerto Rico compared to mothers born in other countries, and this difference increased with older child age (Figure 2). Most children who were aware of their mother's serostatus (70% or 117/166) learned via a planned disclosure by the mother herself. The remainder of the children learned about their mothers’ serostatus through others means, including from a family member, from a non‐family member, by seeing maternal HIV medications and other unplanned disclosures.

### 3.3 Maternal and child characteristics by disclosure status

Table 1 summarises the characteristics of children and their mothers among children who were reported to be aware versus unaware of their mother's HIV serostatus. Compared to mothers whose children were aware of their HIV serostatus, mothers whose children were not aware were more likely to be born outside of the U.S. and Puerto Rico (31% vs. 17%). Mothers of children who were aware of their HIV serostatus were more frequently separated or divorced and had a longer mean number of years since their HIV diagnosis compared to mothers who did not disclose. Children who were aware of their mother's HIV serostatus were more frequently female and in high school versus middle school and elementary school.

**Table 1:** Characteristics of children and biological mothers by disclosure status to child at all visits

	Disclosed (*n*=208)	Did not disclose (*n*=531)	Total (*N*=739)
Cohort	*n* (%)	*n* (%)	*n* (%)
Dynamic	16 (8%)	103 (19%)	119 (16%)
Static	192 (92%)	428 (81%)	620 (84%)
Child sex			
Male	90 (43%)	284 (53%)	374 (51%)
Female	118 (57%)	247 (47%)	365 (49%)
Child visit age (in years)			
11	30 (14%)	235 (44%)	265 (36%)
13	57 (27%)	144 (27%)	201 (27%)
15	65 (31%)	88 (17%)	153 (21%)
17	56 (27%)	64 (12%)	120 (16%)
Child race			
Asian	0 (0%)	1 (0%)	1 (0%)
Black or African American	128 (62%)	322 (61%)	450 (61%)
White	66 (32%)	148 (28%)	214 (29%)
More than one race	5 (2%)	7 (1%)	12 (2%)
Unknown	9 (4%)	53 (10%)	62 (8%)
Child ethnicity			
Hispanic or Latino	63 (30%)	195 (37%)	258 (35%)
Not Hispanic or Latino	144 (69%)	334 (63%)	478 (65%)
Unknown	1 (0%)	2 (0%)	3 (0%)
Child education level			
Grade 6 and younger	45 (22%)	269 (51%)	314 (42%)
Grades 7 and 8	56 (27%)	124 (23%)	180 (24%)
Grades 9 to 12	105 (50%)	134 (25%)	239 (32%)
Other	2 (1%)	4 (1%)	6 (1%)
Child primary language			
English	167 (80%)	395 (74%)	562 (76%)
Spanish	24 (12%)	81 (15%)	105 (14%)
English and another language	16 (8%)	35 (7%)	51 (7%)
Other	1 (0%)	20 (4%)	21 (3%)
Mother's birthplace region			
US and Puerto Rico	170 (82%)	366 (69%)	536 (73%)
Other country	35 (17%)	163 (31%)	198 (27%)
Unknown	3 (1%)	2 (0%)	5 (1%)
Maternal age (in years)			
*N*	208	531	739
# missing	0	0	0
Mean (95% CI)	42.26 (41.38, 43.13)	41.60 (41.08, 42.12)	41.79 (41.34, 42.24)
Min, Max	27.07, 58.03	27.72, 57.13	27.07, 58.03
Median (Q1, Q3)	40.90 (37.72, 47.62)	41.23 (36.95, 46.13)	41.15 (37.22, 46.45)
Maternal race			
Asian	0 (0%)	1 (0%)	1 (0%)
Black or African American	124 (60%)	323 (61%)	447 (60%)
White	77 (37%)	145 (27%)	222 (30%)
American Indian	0 (0%)	1 (0%)	1 (0%)
More than one race	0 (0%)	2 (0%)	2 (0%)
Unknown	7 (3%)	59 (11%)	66 (9%)
Maternal ethnicity			
Hispanic or Latino	66 (32%)	202 (38%)	268 (36%)
Not Hispanic or Latino	142 (68%)	329 (62%)	471 (64%)
Mother's education level			
Less than high school	62 (30%)	174 (33%)	236 (32%)
High school or GED	70 (34%)	152 (29%)	222 (30%)
More than high school	76 (37%)	205 (39%)	281 (38%)
Annual household income			
≤ $10,000	71 (34%)	183 (34%)	254 (34%)
$10,001–$20,000	62 (30%)	113 (21%)	175 (24%)
$20,001–$30,000	30 (14%)	95 (18%)	125 (17%)
≥ $30,001	41 (20%)	133 (25%)	174 (24%)
Unknown	4 (2%)	7 (1%)	11 (1%)
Mother's employment status			
Employed	76 (37%)	245 (46%)	321 (43%)
Not employed	132 (63%)	286 (54%)	418 (57%)
Mother's living arrangement			
Living with partner/spouse	84 (40%)	237 (45%)	321 (43%)
Not living with partner/spouse	122 (59%)	288 (54%)	410 (55%)
Other	2 (1%)	6 (1%)	8 (1%)
Mother's marital status			
Married	50 (24%)	157 (30%)	207 (28%)
Separated/divorced	43 (21%)	62 (12%)	105 (14%)
Widowed	10 (5%)	7 (1%)	17 (2%)
Single, never married	105 (50%)	305 (57%)	410 (55%)
Language spoken at home			
English	160 (77%)	350 (66%)	510 (69%)
Spanish	29 (14%)	100 (19%)	129 (17%)
English and another language	19 (9%)	49 (9%)	68 (9%)
Other	0 (0%)	32 (6%)	32 (4%)
Maternal health limitations			
No limitation	123 (59%)	339 (64%)	462 (63%)
At least one limitation	85 (41%)	190 (36%)	275 (37%)
Unknown	0 (0%)	2 (0%)	2 (0%)
Years since maternal HIV diagnosis			
*N*	172	439	611
# missing	36	92	128
Mean (95% CI)	19.38 (18.63, 20.12)	17.64 (17.19, 18.09)	18.13 (17.74, 18.52)
Min, Max	11.47, 35.08	11.18, 32.62	11.18, 35.08
Median (Q1, Q3)	18.38 (15.61, 22.46)	16.82 (13.66, 20.52)	17.53 (14.22, 21.04)

### 3.4 Reported reasons for disclosing and not disclosing maternal HIV serostatus

For each unique child aware of their mother's HIV serostatus (*n* = 166), the mother was asked about their reasons for disclosure. Reasons that were “very much a factor” in their decision to disclose included: wanting the child to hear it from the mother rather than from someone else (72% or 119/166), not wanting to keep the diagnosis a secret from the child (70% or 117/166), wanting to educate the child about HIV to help the child avoid acquiring HIV (70%) and wanting the child to understand the mother's health condition and/or treatment needs (67% or 111/166). For children who were unaware of their mother's HIV serostatus (*n* = 410), the most frequent reasons identified by mothers as “very much a factor” for not disclosing were not wanting to worry or burden the child with this information (80% or 326/410) and wanting to protect the child so others will not hurt them because of their mother's HIV serostatus (65% or 266/410) (data not shown in tables).

### 3.5 Intention to disclose maternal HIV serostatus

Among the 410 children who were not aware of their mother's HIV serostatus, 16% had mothers who planned to never disclose their diagnosis, 27% who planned to disclose in the near future and 42% who planned to disclose in the future but “no time soon.” For children whose mothers planned to disclose sometime in the future (*n* = 285), the most frequent reasons endorsed as “very much a factor” for wanting to disclose included wanting the child to hear it from them (92%) and wanting to educate the child about HIV (86%).

### 3.6 Distribution of internalised HIV stigma by maternal HIV disclosure status

**Figure 3:**
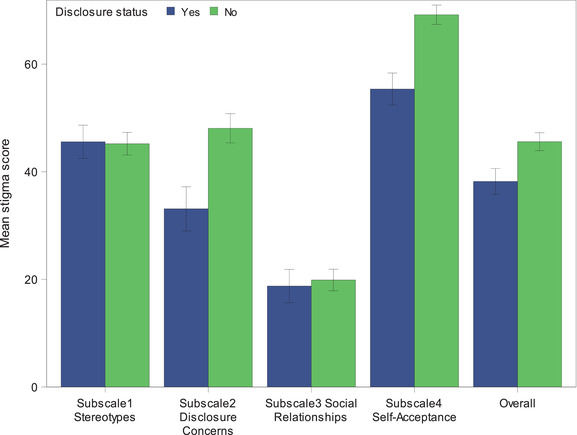
Mean stigma scores overall and for each subscale, by maternal HIV disclosure status. Error bars represent 95% confidence intervals.

The overall mean IHSS score among visits where the mother reported that the child was aware of her HIV serostatus was 38.2 (SD = 17.5), compared to 45.6 (SD = 19.5) among visits where the mother reported that the child was not aware. This trend was seen in all subscales of the IHSS, except for the stereotypes subscale, where mean scores were similar in the two groups (Figure 3). Across all child visit ages, the overall mean IHSS score was lower by at least 5 points for visits where the child was aware of mother's diagnosis.

### 3.7 Associations of internalised HIV stigma with maternal HIV disclosure status

In the unadjusted model, for every 1‐point increase in the overall IHSS score, there was a 2% decrease in the odds of disclosure (OR = 0.98, 95% CI: 0.98, 0.99). This association remained after adjusting for maternal age, marital status and years since maternal HIV diagnosis (adjusted OR [aOR] = 0.985; 95% CI: 0.975, 0.995).

## 4 DISCUSSION

In this study, we found that 29% of CHEU enrolled in SMARTT and whose mothers completed the IHSS were aware of their mother's HIV serostatus. This number was lower than the disclosure proportion reported by Schrimshaw and Siegel, who found that 66% of mothers had disclosed their HIV serostatus to their children who were not living with HIV [14]. Similarly, Abdulrahman et al. reported that 36% of caregivers (both biological and non‐biological parents) had disclosed their HIV status to children living with and without HIV [44]. The prevalence of disclosure in our study was similar to the 28% reported by Armistead et al. whose study population included biological mothers and their CHEU [13]. Differences in disclosure proportions may be due to concomitant stigmas related to mothers’ race, gender, socio‐economic status, immigration status and mental health conditions exacerbating internalised HIV stigma and complicating the disclosure process. In addition, there is no standardised way of measuring disclosure. In our study, we defined disclosure as the child being aware of their mother's HIV serostatus regardless of who informed the child. Other studies define disclosure as the parent or caregiver directly disclosing their HIV serostatus to their children [8, 17, 45, 46].

The reasons for disclosure and non‐disclosure in this study were similar to other studies examining disclosure of maternal HIV serostatus to their children [14, 15, 47]. The primary reasons for mothers disclosing were to ensure that their children learned about their mother's diagnosis directly from them, not wanting to keep the diagnosis a secret and wanting to educate the child about HIV. The main reported reasons for not disclosing were not wanting to burden the child with diagnosis information and wanting to protect the child from being hurt by others.

Most of the mothers in our study had disclosed or planned to disclose their serostatus to their children. Only 16% of mothers reported that they never want to disclose their HIV serostatus to their children. Most reported that their diagnosis was personal. This is understandable given that parents living with HIV experience challenges in managing the disclosure process, discomfort discussing death and reasons for their diagnosis, fear of children's emotional reactions and perceived rejection from children [6]. Since some parents may not have the appropriate resources or skills to facilitate HIV disclosure, specialised intervention programmes, such as Teaching, Raising, and Communication with Kids (TRACK), developed by Murphy et al., may help prepare and support parents dealing with disclosure decisions [47].

In this study, we found a greater likelihood of maternal HIV serostatus disclosure with the child's increasing age. Increased disclosure to older children versus younger children may be attributed to the perceived maturity of the child, the perceived ability of the child to keep the mother's HIV serostatus a secret and needing assistance or support from that child [13, 26, 44, 48]. We also found lower proportions of maternal HIV serostatus disclosure among non‐U.S.‐born mothers compared to U.S.‐ and Puerto Rico‐born, which may be related to community‐level factors [44, 49, 50]. Mothers born in other countries may fear that disclosure to their children may lead to community disclosure and subsequent community shaming and judgements. Non‐U.S.‐born mothers may also be uncomfortable seeking help to disclose to their children from their HIV providers [6]. Additionally, due to traditional gender roles and unique cultural conditioning, some mothers may find it inappropriate or uncomfortable to discuss perceived taboo subjects, such as sex and sexuality [51].

We found that higher levels of internalised HIV stigma were associated with lower odds of disclosure to CHEU, which is consistent with other studies [37, 38, 39, 52, 53, 54]. This study adds to the existing literature on the relationship between internalised HIV stigma and maternal HIV serostatus disclosure to CHEU. It contextualises HIV stigma and maternal disclosure of HIV status from the position of a large, multi‐site, U.S.‐based longitudinal cohort study of mothers and their adolescent CHEU, which is currently missing from the literature. Understanding maternal serostatus disclosure to CHEU and related factors may be helpful for healthcare providers in assisting parents with disclosure‐related matters. For CHEU, knowledge of their mothers’ HIV seropositivity and associated struggles (i.e. navigating complex healthcare systems, ART adherence, stigma and discrimination) and their own exposure to HIV and ART may help them avoid high‐risk behaviours. Knowledge of their own exposure to HIV and ART may also be important for the management of health conditions that may not arise until an individual has advanced into adulthood [41].

There are several study limitations in need of acknowledgement. Due to the cross‐sectional design of the study and that disclosure is a dynamic process, we were unable to explore how stigma leads to disclosure decisions. The IHSS, while validated both in women living with HIV in the U.S. and outside of the U.S., may not have captured the nuanced experiences of mothers living and parenting with HIV who were born in other countries. Those completing the stigma and disclosure assessments were older and living with their HIV diagnosis longer. Therefore, the findings of this study may not be generalizable to younger mothers and mothers with more recent HIV diagnoses, as these groups may experience stigma and disclosure decisions differently. Moreover, stigma assessments were implemented only after a certain time point, and only mothers with children who reached the age of at least 11 years were assessed, limiting the generalizability of study results to mothers with younger children. Additionally, we did not have appropriate maternal data on mental health conditions and intimate partner violence, which may have impacted the internalisation of HIV stigma and decisions around serostatus disclosure to their CHEU.

Importantly, this study found that most mothers (84%) intend to disclose their HIV serostatus to their CHEU. However, these mothers may not have the necessary tools or needed support to do so. Thus, the results of this study have implications for future research and programme development. First, interventions are needed to reduce internalised HIV stigma. Mobilizing and strengthening communities impacted by HIV with opportunities for peer leadership, support, education, outreach and advocacy may be valuable in reducing internalised HIV stigma and its harmful effects [4]. Incorporating disclosure practices that recognise and accommodate the cultural diversity and norms of families may be particularly advantageous for mothers born outside of the U.S [8]. Studies exploring the specific role of HIV stigma experienced in the community and the impact of non‐HIV‐related discrimination among mothers who immigrated to the U.S. on disclosure decisions to children may also provide rich experiential insight for shaping community‐level interventions.

## 5 CONCLUSIONS

The current study explored the prevalence of maternal HIV serostatus disclosure among CHEU and their biological mothers enrolled in the SMARTT study and evaluated the relationship between internalised HIV stigma and disclosure. We found that disclosure was prevalent in 29% of mother‐child pairs and that higher internalised HIV stigma was associated with lower odds of disclosing to the child. An additional 54% of mothers planned to disclose to HIV serostatus in the future. Providing culturally sensitive and unbiased support to women as they make decisions about disclosure of their HIV serostatus to their children may entail addressing internalised HIV stigma and consideration of community‐level factors, particularly for non‐U.S.‐born mothers.

## COMPETING INTERESTS

The authors declare that they have no competing interests.

## AUTHORS’ CONTRIBUTIONS

MD wrote the first draft of the manuscript. DK and JL completed the data analysis and contributed to the revisions of the manuscript. CB, EGC, RS, LS and TF contributed to the revisions of the manuscript. All authors have read and approved the final version of the manuscript.

## FUNDING

The study was supported by the Eunice Kennedy Shriver National Institute of Child Health & Human Development (NICHD), Office of the Director, National Institutes of Health (OD), National Institute of Dental & Craniofacial Research (NIDCR), National Institute of Allergy and Infectious Diseases (NIAID), National Institute of Neurological Disorders and Stroke (NINDS), National Institute on Deafness and Other Communication Disorders (NIDCD), National Institute of Mental Health (NIMH), National Institute of Drug Abuse (NIDA), National Cancer Institute (NCI), National Institute on Alcohol Abuse and Alcoholism (NIAAA), and National Heart, Lung, and Blood Institute (NHLBI) through cooperative agreements with the Harvard T.H. Chan School of Public Health (HD052102) (Principal Investigator: George R Seage III; Program Director: Liz Salomon) and the Tulane University School of Medicine (HD052104) (Principal Investigator: Russell Van Dyke; Co‐Principal Investigator: Ellen Chadwick; Project Director: Patrick Davis) and through Harvard T.H. Chan School of Public Health for the Pediatric HIV/AIDS Cohort Study 2020 (P01HD103133) (Multiple Principal Investigators: Ellen Chadwick, Sonia Hernandez‐Diaz, Jennifer Jao, Paige Williams; Program Director: Liz Salomon). Data management services were provided by Frontier Science (Data Management Center Director: Suzanne Siminski), and regulatory services and logistical support were provided by Westat, Inc (Project Directors: Julie Davidson, Tracy Wolbach).The following institutions, clinical site investigators and staff participated in conducting PHACS SMARTT in 2020, in alphabetical order: **Ann & Robert H. Lurie Children's Hospital of Chicago**: Ellen Chadwick, Margaret Ann Sanders, Kathleen Malee; **Baylor College of Medicine**: Mary Paul, Ruth Eser‐Jose, Chivon McMullen‐Jackson, Lynnette Harris; **BronxCare Health System**: Murli Purswani, Mahoobullah Mirza Baig, Alma Villegas, Marvin Alvarado; **Children's Diagnostic & Treatment Center**: Lisa‐Gaye Robinson, Jawara Dia Cooley, James Blood, Patricia Garvie; **New York University School of Medicine**: William Borkowsky, Nagamah Sandra Deygoo, Jennifer Lewis; **Rutgers—New Jersey Medical School**: Arry Dieudonne, Linda Bettica, Juliette Johnson, Karen Surowiec; **St. Jude Children's Research Hospital**: Katherine Knapp, Jamie Russell‐Bell, Megan Wilkins, Stephanie Love; **San Juan Hospital Research Unit/Department of Pediatrics, San Juan Puerto Rico**: Nicolas Rosario, Lourdes Angeli‐Nieves, Vivian Olivera; **SUNY Downstate Medical Center**: Stephan Kohlhoff, Ava Dennie, Jean Kaye, Jenny Wallier; **Tulane University School of Medicine**: Karen Craig, Margarita Silio, Patricia Sirois; **University of Alabama, Birmingham**: Cecelia Hutto, Paige Hickman, Julie Huldtquist, Dan Marullo; **University of California, San Diego**: Stephen A. Spector, Veronica Figueroa, Megan Loughran, Sharon Nichols; **University of Colorado, Denver**: Elizabeth McFarland, Christine Kwon, Carrie Glenny, Jennifer Englund; **University of Florida, Center for HIV/AIDS Research, Education and Service**: Mobeen Rathore, Saniyyah Mahmoudi, Sarah El‐Hassan, Jamilah Tejan; **University of Illinois, Chicago**: Karen Hayani, Lourdes Richardson, Renee Smith, Alina Miller; **University of Miami**: Gwendolyn Scott, Gustavo Gil Garcia, Gabriel Fernandez, Anai Cuadra; **Keck Medicine of the University of Southern California**: Toni Frederick, Mariam Davtyan, Guadalupe Morales‐Avendano; **University of Puerto Rico School of Medicine, Medical Science Campus**: Zoe M. Rodriguez, Lizmarie Torres, Nydia Scalley.

The Pediatric HIV/AIDS Cohort Study (PHACS) network was supported by the *Eunice Kennedy Shriver* National Institute of Child Health & Human Development (NICHD), Office of The Director, National Institutes of Health (OD), National Institute of Dental & Craniofacial Research (NIDCR), National Institute of Allergy and Infectious Diseases (NIAID), National Institute of Neurological Disorders and Stroke (NINDS), National Institute on Deafness and Other Communication Disorders (NIDCD), National Institute of Mental Health (NIMH), National Institute on Drug Abuse (NIDA), National Cancer Institute (NCI), National Institute on Alcohol Abuse and Alcoholism (NIAAA), and the National Heart, Lung, and Blood Institute (NHLBI) through cooperative agreements with the Harvard T.H. Chan School of Public Health (HD052102), Tulane University School of Medicine (HD052104) and Harvard T.H. Chan School of Public Health for the Pediatric HIV/AIDS Cohort Study 2020 network (P01HD103133).

## DISCLAIMER

The conclusions and opinions expressed in this article are those of the authors and do not necessarily reflect those of the National Institutes of Health or the U.S. Department of Health and Human Services.

## Data Availability

The data that support the findings of this study can be made available upon reasonable request.
